# Detecting Mandible Fractures in CBCT Scans Using a 3-Stage Neural Network

**DOI:** 10.1177/00220345241256618

**Published:** 2024-06-24

**Authors:** N. van Nistelrooij, S. Schitter, P. van Lierop, K. El Ghoul, D. König, M. Hanisch, A. Tel, T. Xi, D.G.E. Thiem, R. Smeets, L. Dubois, T. Flügge, B. van Ginneken, S. Bergé, S. Vinayahalingam

**Affiliations:** 1 Department of Oral and Maxillofacial Surgery, Radboud University Medical Center, Nijmegen, The Netherlands; 2 Charité – Universitätsmedizin Berlin, corporate member of Freie Universität Berlin and Humboldt-Universität zu Berlin, Department of Oral and Maxillofacial Surgery, Berlin, Germany; 3 Department of Oral and Maxillofacial Surgery, Division of Regenerative, Orofacial Medicine, University Medical Center Hamburg-Eppendorf, Hamburg, Germany; 4 Department of Oral and Maxillofacial Surgery, Erasmus Medical Center, Rotterdam, The Netherlands; 5 Department of Oral and Maxillofacial Surgery, Universitätsklinikum, Münster, Münster, Germany; 6 Clinic of Maxillofacial Surgery, Head-Neck and NeuroScience Department University Hospital of Udine, Udine, Italy; 7 Department of Oral and Maxillofacial Surgery, Facial Plastic Surgery, University Medical Centre Mainz, Mainz, Germany; 8 Department of Oral and Maxillofacial Surgery, Amsterdam UMC, University of Amsterdam, Amsterdam, The Netherlands; 9 Diagnostic Image Analysis Group, Radboud University Medical Center, Nijmegen, The Netherlands

**Keywords:** cone-beam computed tomography, deep learning, maxillofacial surgery, artificial intelligence, mandibular fractures, open source software

## Abstract

After nasal bone fractures, fractures of the mandible are the most frequently encountered injuries of the facial skeleton. Accurate identification of fracture locations is critical for effectively managing these injuries. To address this need, JawFracNet, an innovative artificial intelligence method, has been developed to enable automated detection of mandibular fractures in cone-beam computed tomography (CBCT) scans. JawFracNet employs a 3-stage neural network model that processes 3-dimensional patches from a CBCT scan. Stage 1 predicts a segmentation mask of the mandible in a patch, which is subsequently used in stage 2 to predict a segmentation of the fractures and in stage 3 to classify whether the patch contains any fracture. The final output of JawFracNet is the fracture segmentation of the entire scan, obtained by aggregating and unifying voxel-level and patch-level predictions. A total of 164 CBCT scans without mandibular fractures and 171 CBCT scans with mandibular fractures were included in this study. Evaluation of JawFracNet demonstrated a precision of 0.978 and a sensitivity of 0.956 in detecting mandibular fractures. The current study proposes the first benchmark for mandibular fracture detection in CBCT scans. Straightforward replication is promoted by publicly sharing the code and providing access to JawFracNet on grand-challenge.org.

## Introduction

Mandibular fractures represent a significant portion of facial traumas, accounting for at least 25% of all facial fracture cases (Iida et al. 2001; Ahmad et al. 2014). The primary causes of these fractures include motor vehicle accidents, assaults, sports injuries, and falls (Afrooz et al. 2015). Treatment varies depending on the severity of the fracture. They can include noninterventional expectative management, conservative management by maxillomandibular fixation, or surgical management by open reduction and internal fixation (Pickrell et al. 2017). Ensuring an accurate diagnosis and a well-considered treatment plan is crucial for restoring occlusion, anatomy, function, and esthetics (Stacey et al. 2006).

Recently, the use of cone-beam computed tomography (CBCT) for diagnosing mandibular fractures has increased significantly (Patel et al. 2019). CBCT provides an accurate 3-dimensional (3D) visualization of the facial skeleton and is more effective at detecting mandibular fractures than 2-dimensional (2D) panoramic radiographs (PRs; Kobayashi-Velasco et al. 2017). In addition, CBCT exposes patients to lower radiation levels compared with computed tomography (CT; Loubele et al. 2009). However, the lower radiation dose increases noise and less distinct boundaries between different tissues. As subtle changes in bone density often mark fracture lines, the intrinsic characteristics of CBCT make it challenging to detect fractures (Loubele et al. 2007; Yu et al. 2010). Consequently, analyzing a scan for fractures can take up to 5 min (Jin et al. 2020). This time-consuming radiographic examination increases the workload of the oral and maxillofacial (OMF) radiologist, resulting in decreased diagnostic performance due to fatigue and visual strain (Krupinski et al. 2010). An automated assistance system may provide reliable, fast, consistent, and accurate support in the assessment of mandibular fractures, in particular for less experienced professionals (Thrall et al. 2018; Guermazi et al. 2021). The aid of an automated system during radiographic examination can reduce analysis time and missed fractures, a preventable cause of potential litigation costs and patient morbidity (Canoni-Meynet et al. 2022).

Artificial intelligence (AI) and deep learning have been introduced in dentistry, leading to significant changes in the digital workflow (Ding et al. 2023; Mahdi et al. 2023). Deep learning algorithms such as convolutional neural networks (CNNs) process input data (e.g., images) to outputs (e.g., disease present/absent) while learning progressively from higher-level features of the input data (Mohammad-Rahimi et al. 2023). Different models have been proposed to support clinicians in efficient, accurate, and reliable diagnosis (Kuo et al. 2022; Wang et al. 2022), including the detection of mandibular fractures (Sehat et al. 2023). For example, several methods have been proposed for PRs based on image classification (Nishiyama et al. 2021; Warin et al. 2022), object detection (Son et al. 2021; Vinayahalingam et al. 2022; Warin et al. 2022), including in combination with semantic segmentation (Son et al. 2022; Shahnavazi and Mohamadrahimi 2023). In addition, 2 methods for CT scans have been introduced that operate on axial slices (Warin et al. 2023) and 2D projections (Wang et al. 2022). However, no study to date has evaluated the detection of mandibular fractures on CBCT scans using deep learning. In addition, the development of robust and trustworthy computational models to detect maxillofacial and mandibular fractures is limited due to missing open-source code (Mörch et al. 2021). Therefore, AI studies in OMF surgery are often subject to methodological and reporting limitations that directly affect their transparency and replicability.

In this study, a 3-stage neural network dubbed JawFracNet is proposed and evaluated. The aim is to provide a benchmark for the automated detection of mandibular fractures in CBCT scans for continued development of diagnostic systems. The code of JawFracNet is publicly accessible at github.com/nnistelrooij/jawfrac. Furthermore, JawFracNet is available online at grand-challenge.org/algorithms/jawfracnet.

## Materials and Methods

This retrospective diagnostic accuracy study was conducted following the code of ethics of the World Medical Association (Declaration of Helsinki). The Institutional Review Board approved this study and the use of patient data (Medical Association of Hamburg; case No. 2022-300184-WF). The checklist for AI research in dentistry has been consulted for reporting (Schwendicke et al. 2021).

### Data

The current study includes a convenience sample of 164 CBCT scans with nonfractured, complete mandibles and 171 CBCT scans with mandibular fractures obtained from the Department of Oral and Maxillofacial Surgery at the University Medical Center Hamburg-Eppendorf between July 2012 and September 2022. Each patient contributed 1 CBCT scan, resulting in a prevalence of mandibular fractures of 51%. Written informed consent was collected from each patient, and all patient data were pseudonymized prior to analysis. Patient age ranged from 17 to 92 y, with a median and interquartile range of 33 and 32 y, respectively (Table 1). Scans with unknown voxel spacing, visual artifacts, or osteolytic lesions were excluded. The collected CBCT scans were converted to NIfTI format.

**Table 1.** Patients with Mandibular Fractures Included in the Current Study.^a^

	Patients	%
Gender		
Male	122	71
Female	49	29
Mandible in FOV		
Not occluded	139	81
Partially occluded	24	14
Mostly occluded	8	4.7
Fracture types		
Nondisplaced	111	65
Displaced	43	25
Both	17	9.9
Fracture counts		
1	107	63
2	50	29
≥3	14	8.2
	Median (IQR)	Range
Age, y		
Male	31 (28)	17–92
Female	46 (40)	17–87
	Fractures	%
Fracture location		
Median	23	9.2
Paramedian	49	20
Angulus	50	20
Ramus	76	30
Coronoid	4	1.6
Condyle	49	20
Fracture type		
Nondisplaced	180	72
Displaced	71	28

FOV, field of view; IQR, interquartile range.

a Males were overrepresented in the included patients, and nondisplaced fractures occurred more often than displaced fractures did. A fracture occurred most often in the ramus subregion and least often in the median and coronoid subregions.

### Data Annotation

The complete mandibles and mandibular fractures were segmented slice by slice based on electronic medical records by a resident in OMF surgery (S.S., 4 y experience) and reviewed and revised by an experienced OMF surgeon (T.X., minimum 15 y experience) using ITK-SNAP (version 3.6.0; Appendix Fig. 1A–D). Annotated scans were divided into 3 sets: 60% for training, 20% for validation, and 20% for testing. This division was performed while considering the stratification based on the subregion of each fracture and whether the fracture was displaced. To ensure the validity of the test set, each test scan was independently evaluated for the detection of mandibular fractures by 3 OMF surgeons (P.v.L., M.H., A.T.). Any discrepancies were examined by an experienced OMF surgeon (T.X.) to reach a final consensus.

### Deep Learning Model

**Figure 1.**
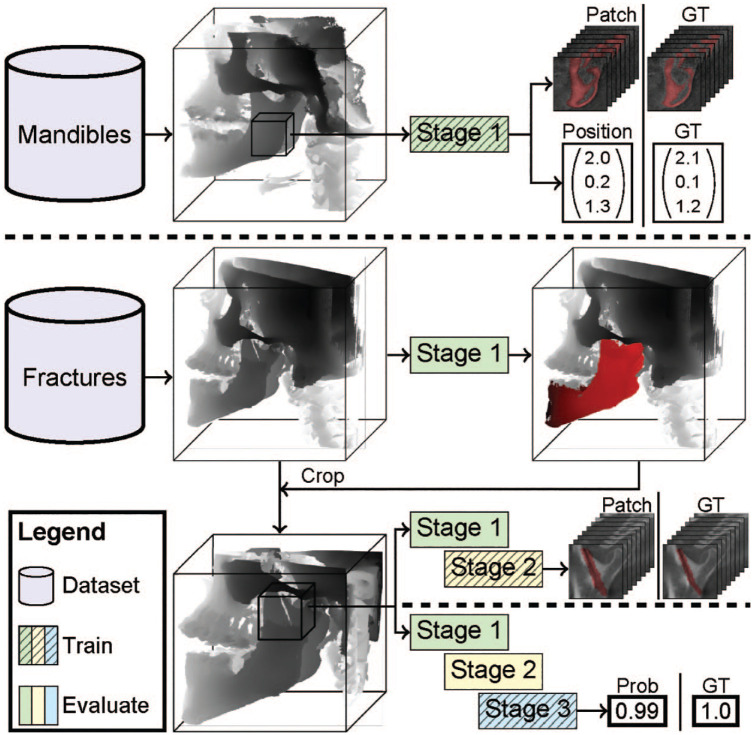
JawFracNet successively trains 3 stages. First, the scans with complete mandibles are used to train stage 1 to predict a mandible segmentation and a patch position. Next, scans with fractures are cropped around the mandible segmentation. Stage 2 is trained to predict a segmentation of nondisplaced fractures. Lastly, stage 3 is trained to classify whether a patch contains any fracture. Prob, probability; GT, ground truth.

A model composed of 3 stages is proposed (Fig. 1). These stages are responsible for predicting the mandible segmentation, fracture segmentation, and fracture classification, respectively. Predictions generated from all patches were combined to create segmentations of the input scan. Subsequently, postprocessing steps were applied to filter out irrelevant segmentations and consolidate the predicted fractures to obtain the final result.

#### Stage 1: Mandible segmentation

The initial step of the model involved mandible segmentation within the CBCT scan. Patches measuring 64 × 64 × 64 were extracted from the scan, and 3 additional input channels were created using the grayscale adaptive perception module. This module constrained the intensity values of each patch within 3 adaptive ranges (Liu et al. 2022). The patches were subsequently processed with an encoder, global average pooling (GAP), and 2 fully connected layers to predict the relative position of the patch, which was supervised by the smooth L1 loss function. The mandible segmentation was predicted by a U-Net with a shared encoder (Appendix Fig. 2, green box) and supervised using binary cross-entropy (BCE).

#### Stage 2: Fracture segmentation

The scan was cropped accordingly following the predicted mandible segmentation. Subsequently, in stage 2, the model focused on segmenting the fracture lines, specifically for nondisplaced fractures, shown in Figure 1. The decoder features and segmentation logits of a patch in stage 1 were channel-wise concatenated to provide a patch with more context to stage 2 (Appendix Fig. 2, green box). This patch was processed by a cascade of 2 U-Nets, in which the second U-Net improved the segmentation logits provided by the first U-Net (Appendix Fig. 2, yellow box). Both U-Nets were supervised by BCE. Class imbalance was addressed by sampling patches with nondisplaced fractures and patches without fractures at an equal frequency. Furthermore, the fracture annotations were morphologically dilated and Gaussian filtered to increase the number of positive voxels.

#### Stage 3: Fracture classification

The final stage predicted whether an input patch contained any fracture (Appendix Fig. 2, blue box). The decoder features and segmentation logits of stage 1 and the segmentation logits of stage 2 were channel-wise concatenated to provide a patch with more context. This patch was processed by an encoder, GAP, and 2 fully connected layers to predict whether the patch contained any fracture, which was supervised using BCE. No distinction was made between nondisplaced and displaced fractures.

During inference, classification probabilities generated by stage 3 were interpolated to cover all voxels. Afterward, connected component analysis determined fracture proposals based on the voxel-level segmentations from stage 2 and stage 3. The final result was determined by uniting the fracture proposals from both stage 2 and stage 3. In this way, JawFracNet combined the high segmentation precision from stage 2 with the high detection sensitivity from stage 3.

### Model Training

The model was implemented using PyTorch Lightning (version 1.6.5) based on PyTorch 1.12.0 (Paszke et al. 2019), in which the U-Net architecture (Appendix Fig. 2, red box) was adopted from the study of Jin et al. (2020). Each hidden layer was followed by batch normalization and a leaky ReLU (Ioffe and Szegedy 2015; Xu et al. 2015). The AdamW optimizer was used for training with a weight decay of 0.01 (Loshchilov and Hutter 2017). Each stage was successively trained for a maximum of 500 epochs with a mini-batch size of 4. Stage 1 was trained with a learning rate of 0.005, whereas stages 2 and 3 were trained with a learning rate of 0.002. A cosine annealing schedule with linear warmup was used to adapt the learning rate. Training and inference were done on a workstation with an RTX A6000 48 GB and 128 GB memory.

### Model Evaluation

The mandible segmentations of JawFracNet were evaluated by comparing predicted and annotated segmentations of complete mandibles using Dice coefficient $=\frac{2TP}{2TP+FP+FN}$ and intersection over union (IoU) $=\frac{TP}{TP+FP+FN}$ , where *TP*, *FP*, and *FN* represent true-positive, false-positive, and false-negative voxels, respectively. Predicted fracture proposals were compared with the ground-truth fracture annotations to determine *TP*, *FP*, and *FN* fracture-level detections in the test scans with mandibular fractures. Fracture proposals with a low confidence based on their average segmentation probability were excluded to reduce false-positive detections. The detections were used to report precision $=\frac{TP}{TP+FP}$ and sensitivity $=\frac{TP}{TP+FN}$ for all fractures and for nondisplaced and displaced fractures, separately. In addition, a free-response receiver-operating characteristic (FROC) curve was determined by computing the precision and sensitivity for all possible operating points. The FROC curve was summarized as the area under the curve (AUC) by averaging the sensitivities for 1/16, 1/8, 1/4, 1/2, and 1 false-positive detection per scan.

In addition, JawFracNet was compared with 3 OMF surgeons (R1, R2, R3) regarding effectiveness and efficiency. Three OMF surgeons were included to detect mandibular fractures in the test scans. The surgeons were instructed and calibrated using scans outside the test split, and they identified mandibular fractures based solely on the CBCT scan. Furthermore, scans were presented to each surgeon randomly to limit biases due to presentation order, and 50% of the scans did not contain fractures. A surgeon indicated a fracture by a coarse segmentation on 1 slice while recording their analysis time. The difference between the analysis times of JawFracNet and the OMF surgeons was determined using the Kruskal-Wallis H test, and pairwise comparisons were performed using the Wilcoxon signed-rank test. Statistical significance was defined as .

## Results

### Mandible segmentation

**Figure 2.**
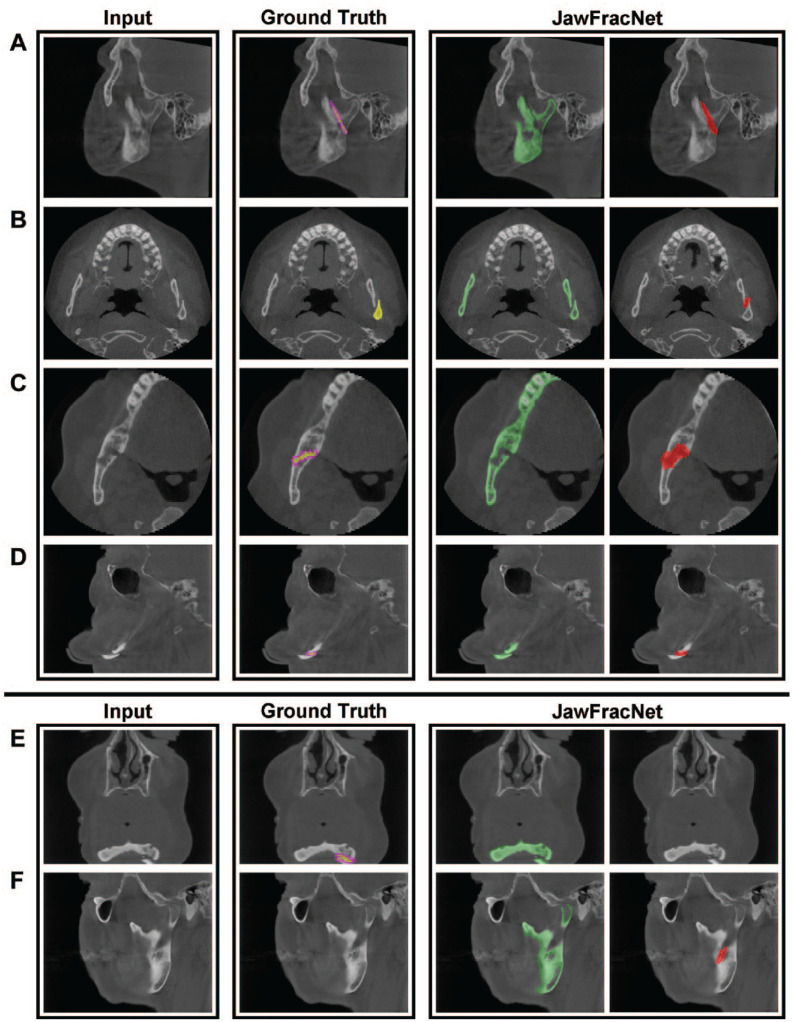
Representative results of JawFracNet for a nondisplaced fracture (**A**), a displaced fracture (**B**), an incomplete mandible (**C**), and a patient with a resected maxilla (**D**). Two failure cases are shown with a false-negative (**E**) and false-positive (**F**) fracture detection.

JawFracNet was effective for segmentation of complete mandibles (Dice = 0.904, IoU = 0.824). Furthermore, the qualitative results for fractured mandibles highlight the precise segmentation of the mandible without teeth, fracture lines, or cancellous bone (Fig. 2).

### Fracture Detection

**Figure 3.**
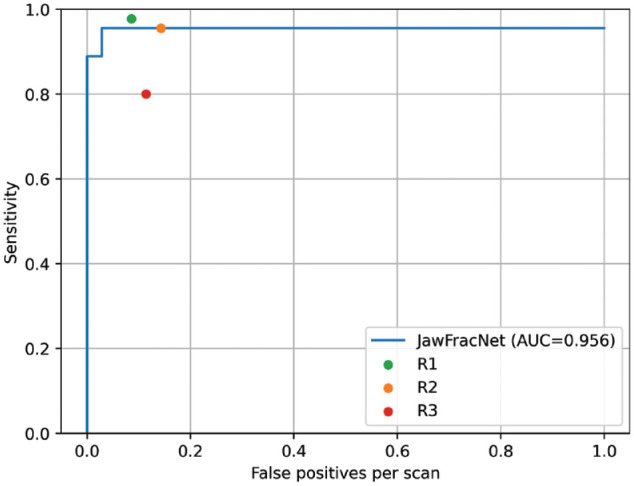
A free-response receiver-operating characteristic (FROC) analysis was performed that visualizes the effectiveness of a detection model by plotting sensitivity against the number of false-positive detections (misdiagnoses) per scan for all confidence scores. The confidence was determined as the average segmentation probability of a fracture proposal. The area under the curve (AUC) was determined by averaging the sensitivity at 1/16, 1/8, 1/4, 1/2, and 1 false-positive detection per scan.

To assess the performance of JawFracNet, the fracture predictions were compared with the ground-truth fractures in the held-out test set to determine the true-positive, false-positive, and false-negative results. Based on 45 fractures in 35 test scans, JawFracNet achieved a high precision of 0.978 and a sensitivity of 0.956 in fracture detection. However, it failed to detect 1 nondisplaced and 1 displaced fracture (Table 2). In addition, 1 false-positive fracture was generated by JawFracNet. A consistently high sensitivity was observed when choosing different operating points resulting in an AUC of 0.956, indicating the robustness of JawFracNet for detecting mandibular fractures (Fig. 3).

**Table 2.** Fracture Detection and Analysis Times of JawFracNet and OMF Surgeons.^a^

	Nondisplaced	Displaced	Total		Seconds	
Participant	Sensitivity	Sensitivity	Precision	Sensitivity	Mean ± SD	*P* Value
JawFracNet	**0.971**	0.917	**0.978**	0.957	39.4 ± 14.4	
R1	**0.971**	**1.0**	0.938	**0.978**	66.5 ± 21.4	<0.001**
R2	0.941	**1.0**	0.902	0.957	70.5 ± 20.3	<0.001**
R3	0.735	0.917	0.891	0.783	93.6 ± 43.0	<0.001**

OMF, oral and maxillofacial; SD, standard deviation.

a Metrics were computed on the test split of the fractures data set, which included 45 fractures. The best metrics are presented in bold. Precision metrics could not be computed for nondisplaced and displaced fractures separately, as the current method and OMF surgeons detected mandibular fractures without specifying the type of fracture. Each time was determined on the test split of the fractures data set with mean and SD determined over scans.

** *P* value was based on a Wilcoxon signed-rank test.

Figure 2A–D shows 4 representative cases with correct fracture detections. Large differences between the manual fracture segmentations and automated fracture segmentations can be observed. Nevertheless, JawFracNet was able to confidently detect all fractures.

Figure 2E and F display 2 illustrative cases of a false-negative and a false-positive outcome, respectively. In the first case, the mandible was partially outside the scan area. A fracture prediction was positioned over the mandibular foramen in the second case. This region, representing an opening on the medial side of the mandibular ramus, was erroneously identified as a fracture.

### Benchmarking JawFracNet with Experts

JawFracNet’s performance and efficiency were benchmarked with OMF surgeons. In general, R1 demonstrated a higher effectiveness than JawFracNet, whereas R3 exhibited a lower effectiveness (Table 2, Fig. 2).

JawFracNet detected fractures in 39.1 ± 14.4 s, whereas the OMF surgeons took more than 1 min (Table 2). The statistical analysis revealed significant differences across the analysis times of JawFracNet and the OMF surgeons (39.1 vs. 76.9, *P* < 0.001). Furthermore, JawFracNet was significantly faster than the OMF surgeons (39.1 s vs. 66.5, 70.5, and 93.6 s, *P* < 0.001).

## Discussion

This study aimed to develop and evaluate an automated approach for detecting mandibular fractures in CBCT scans. To achieve this goal, a 3-stage deep learning model called JawFracNet was developed, achieving a precision of 0.978 and a sensitivity of 0.956. In addition, JawFracNet demonstrated similar effectiveness to OMF surgeons, offering a significant speed advantage.

We believe this study represents the first attempt at automating the detection of facial fractures in CBCT scans. Mandibular fracture detection in CT scans is the gold standard, achieving high sensitivity with experienced radiologists (Naeem et al. 2017). In contrast, due to the relatively less distinct tissue boundaries, mandibular fracture detection in CBCT scans is more challenging (Kobayashi-Velasco et al. 2017). Nonetheless, CBCT is preferred over CT due to the reduced radiation exposure while maintaining diagnostic accuracy. Consequently, an automated method can support radiologists in improving their diagnostic accuracy on CBCT scans, aiming for an accuracy comparable to that of CT scans. The prevalence of mandibular fractures in this study (51%) is within the reported range among facial trauma patients (42.5%–56.9%; Iida et al. 2001; Carvalho et al. 2010; Zamboni et al. 2017). From this perspective, similar results of JawFracNet are expected for the target population.

Two studies have recently applied CNNs to detect mandibular fractures in CT scans. Wang et al. (2022) first transformed a CT scan into a PR. A 2D U-Net predicted a multiclass segmentation of 9 subregions of the mandible based on these PRs. Given the subregion segmentations, 9 image patches were sampled from each straightened CT slice. A ResNet model then classified whether each image patch contained a fracture. The final classification of the subregions was determined by aggregating predictions over all slices. The model was trained on 222 scans, and evaluation on 408 held-out scans revealed a precision and sensitivity of 0.914 and 0.941, respectively.

In comparison, Warin et al. (2023) used a combination of image classification models and object detection models to detect fracture lines in CT images. The authors manually selected the axial view of the maxillofacial bone window to develop the CNN models. The precision and sensitivity for the most effective classification model were 0.60 and 0.53, respectively, with the most effective object detection model achieving a precision and sensitivity of 0.86 and 0.81, respectively. Compared with both studies, JawFracNet achieved superior effectiveness with a precision and sensitivity of 0.978 and 0.956, respectively.

Volumetric segmentations of fractures have been proposed for the automated detection of rib fractures in CT scans (Jin et al. 2020; Yang et al. 2024). The authors employed a similar approach as JawFracNet that predicted a segmentation of the ribs, followed by rib fracture detection. A collection of 660 CT scans was used, and a maximum sensitivity of 0.922 at a precision of 0.546 was reported. The lower effectiveness of this method compared with JawFracNet can be explained by the different domain. The number of rib fractures per CT scan was higher than the number of mandibular fractures per CBCT scan (8.0 vs. 1.3), and more rib fracture types are presented (buckle, nondisplaced, displaced, segmental). Therefore, the results of JawFracNet are likely not representative for fracture detection of other bones.

In the current study, JawFracNet demonstrated performance on par with clinicians (precision of 0.978 and sensitivity of 0.956). JawFracNet missed a displaced mandibular fracture in a scan where the mandible was partially outside the scan area (Fig. 3E). This may be explained by the lower effectiveness of AI models for detecting fractures in the periphery of a patch, due to a lack of context. JawFracNet also generated an erroneous fracture detection over the mandibular foramen (Fig. 3F). A possible explanation for the model’s confusion is the ambiguous start of the mandibular canal.

The proposed method was 2 to 3 times faster than the clinicians. Another advantage is the elimination of observer-dependent errors due to limited concentration, as deep learning models are consistent when presented with the same input data. In line with Open Science practices in AI-driven maxillofacial surgery, the JawFracNet code is publicly available. Furthermore, JawFracNet is available online for testing on in-house CBCT scans. This Open Science approach sets a precedent for AI research in OMF surgery to make replication and reuse of proposed methods easier and faster. Straightforward replication promotes the direct comparison of methods, speeding up the development of more effective and efficient automated solutions for supporting clinical tasks.

A limitation of the current study is the relatively small number of patients with mandibular fractures (Sehat et al. 2023). A CBCT scan is not typically used as a diagnostic tool for the radiographic assessment of a suspected mandibular fracture. Therefore, fewer CBCT scans with mandibular fractures are acquired compared with PRs and CT scans, limiting the current sample size. Furthermore, all CBCT scans came from 1 medical center. The reported results may not indicate JawFracNet’s effectiveness for different scanners or underrepresented fracture types. The current work can be extended by collecting CBCT scans with mandibular fractures from other medical centers. An external validation can be conducted to assess the effectiveness of JawFracNet for scans from another center, which may reveal a decrease in its effectiveness due to a different patient population or variations in imaging equipment and techniques. Therefore, the train data set can be expanded to improve the robustness and generalizability of JawFracNet for novel environments. Another future direction is informing fracture detection with a segmentation of the mandibular canal, possibly resolving the false-positive outcome in Figure 2F (Issa et al. 2022). Moreover, JawFracNet can be extended to classify a fracture detection according to the presence of fracture displacement. This information is crucial for clinical decision making, as different management approaches are indicated for each fracture type. This functionality may be implemented by expanding the fracture classification stage with multiclass classification. In addition, the mandible segmentation stage may be implemented as instance segmentation to predict separate mandibular bone segments, in which a displaced fracture is identified between 2 bone segments. Lastly, a prospective study should be conducted to investigate the clinical benefit of JawFracNet in a collaborative setting where a clinician is informed by the model during analysis.

## Conclusions

JawFracNet provides the first benchmark for mandibular fracture detection in CBCT scans. The current study followed the principles of Open Science by publicly sharing the developed code for straightforward replication. Furthermore, easy access to the method has been enabled through the online availability of the algorithm.

## Author Contributions

N. van Nistelrooij, contributed to conception and design, data acquisition, analysis, and interpretation, drafted the manuscript; S. Schitter, contributed to conception, data acquisition, critically revised the manuscript; P. van Lierop, contributed to conception, data analysis and interpretation, critically revised the manuscript; K. El Ghoul, contributed to design, data interpretation, critically revised the manuscript; D. König, contributed to data acquisition, critically revised the manuscript; M. Hanisch, A. Tel, D. Thiem, contributed to conception, data analysis, critically revised manuscript; T. Xi, T. Flügge, B. van Ginneken, S. Bergé, S. Vinayahalingam, contributed to conception and design, data acquisition, analysis, and interpretation, critically revised the manuscript; R. Smeets, contributed to conception, data acquisition, critically revised the manuscript; L. Dubois, contributed to conception, data analysis and interpretation, critically revised the manuscript. All authors gave their final approval and agree to be accountable for all aspects of the work.

## Supplemental Material

sj-docx-1-jdr-10.1177_00220345241256618 – Supplemental material for Detecting Mandible Fractures in CBCT Scans Using a 3-Stage Neural Network Supplemental material, sj-docx-1-jdr-10.1177_00220345241256618 for Detecting Mandible Fractures in CBCT Scans Using a 3-Stage Neural Network by N. van Nistelrooij, S. Schitter, P. van Lierop, K. El Ghoul, D. König, M. Hanisch, A. Tel, T. Xi, D.G.E. Thiem, R. Smeets, L. Dubois, T. Flügge, B. van Ginneken, S. Bergé and S. Vinayahalingam in Journal of Dental Research

sj-pdf-2-jdr-10.1177_00220345241256618 – Supplemental material for Detecting Mandible Fractures in CBCT Scans Using a 3-Stage Neural Network Supplemental material, sj-pdf-2-jdr-10.1177_00220345241256618 for Detecting Mandible Fractures in CBCT Scans Using a 3-Stage Neural Network by N. van Nistelrooij, S. Schitter, P. van Lierop, K. El Ghoul, D. König, M. Hanisch, A. Tel, T. Xi, D.G.E. Thiem, R. Smeets, L. Dubois, T. Flügge, B. van Ginneken, S. Bergé and S. Vinayahalingam in Journal of Dental Research

sj-pdf-3-jdr-10.1177_00220345241256618 – Supplemental material for Detecting Mandible Fractures in CBCT Scans Using a 3-Stage Neural Network Supplemental material, sj-pdf-3-jdr-10.1177_00220345241256618 for Detecting Mandible Fractures in CBCT Scans Using a 3-Stage Neural Network by N. van Nistelrooij, S. Schitter, P. van Lierop, K. El Ghoul, D. König, M. Hanisch, A. Tel, T. Xi, D.G.E. Thiem, R. Smeets, L. Dubois, T. Flügge, B. van Ginneken, S. Bergé and S. Vinayahalingam in Journal of Dental Research

sj-png-4-jdr-10.1177_00220345241256618 – Supplemental material for Detecting Mandible Fractures in CBCT Scans Using a 3-Stage Neural Network Supplemental material, sj-png-4-jdr-10.1177_00220345241256618 for Detecting Mandible Fractures in CBCT Scans Using a 3-Stage Neural Network by N. van Nistelrooij, S. Schitter, P. van Lierop, K. El Ghoul, D. König, M. Hanisch, A. Tel, T. Xi, D.G.E. Thiem, R. Smeets, L. Dubois, T. Flügge, B. van Ginneken, S. Bergé and S. Vinayahalingam in Journal of Dental Research
