# Supplementary material for: Detecting Mandible Fractures in CBCT Scans Using a 3-Stage Neural Network
Source: J Dent Res. 2024 Jun 24;103(13):1384–91. doi: 10.1177/00220345241256618 (PMC11633064; doi:10.1177/00220345241256618)
Supplement: sj-docx-1-jdr-10.1177_00220345241256618 – Supplemental material for Detecting Mandible Fractures in CBCT Scans Using a 3-Stage Neural Network [file sj-docx-1-jdr-10.1177_00220345241256618.docx]

Detecting Mandible Fractures in CBCT Scans using Three-Stage Neural Network

Niels van Nistelrooij, Sophie Schitter, Pieter van Lierop, Khalid El Ghoul, Daniela König, Marcel Hanisch, Alessandro Tel, Tong Xi, Daniel Thiem, Ralf Smeets, Leander Dubois, Tabea Flügge, Bram van Ginneken, Stefaan Bergé, Shankeeth Vinayahalingam

## Annotations


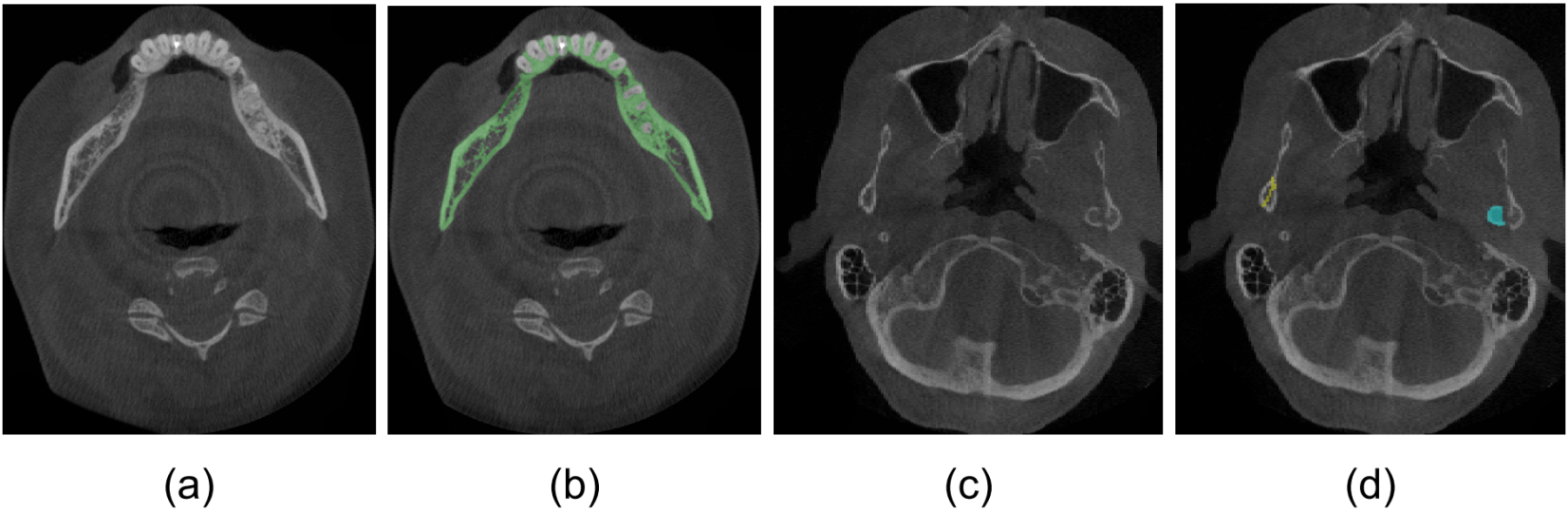


**Appendix Figure 1:** Axial slice of head CBCT scan (a/c) and corresponding segmentation (b/c). The annotation of a complete mandible (b, green) does not include cancellous bone and teeth. Conversely, the annotation of a non-displaced mandibular fracture (d, yellow) precisely follows the fracture line, whereas the annotation of a displaced fracture (d, cyan) includes the complete displaced bone segment.

## Model architecture


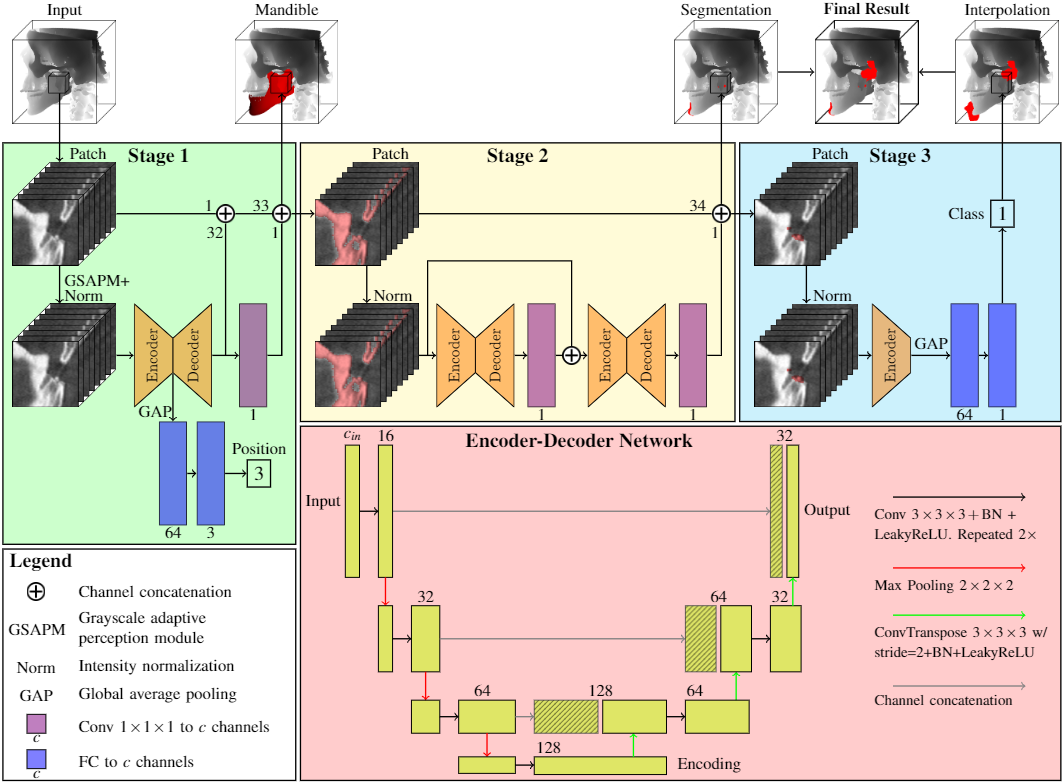


**Appendix Figure 2:** Model architecture of JawFracNet. From the input CBCT scan, patches are sampled and processed by three stages. Stage 1 predicts the relative position of the patch and its mandible segmentation. It then concatenates the decoder features and segmentation logits to the input features. Stage 2 predicts the segmentation of non-displaced fractures using a cascade of encoder- decoder networks. The resulting logits are concatenated to the input features. Stage 3 predicts whether a patch contains any fracture. The sparse logits are then interpolated to the input scan to form a voxel-level segmentation. Lastly, the fractures from stage 2 and stage 3 are united for the final result. The encoder-decoder network represented as orange trapezoids is a 3D convolutional U-Net, shown at the bottom-right (Jin et al. 2020).

## Fracture detection

##
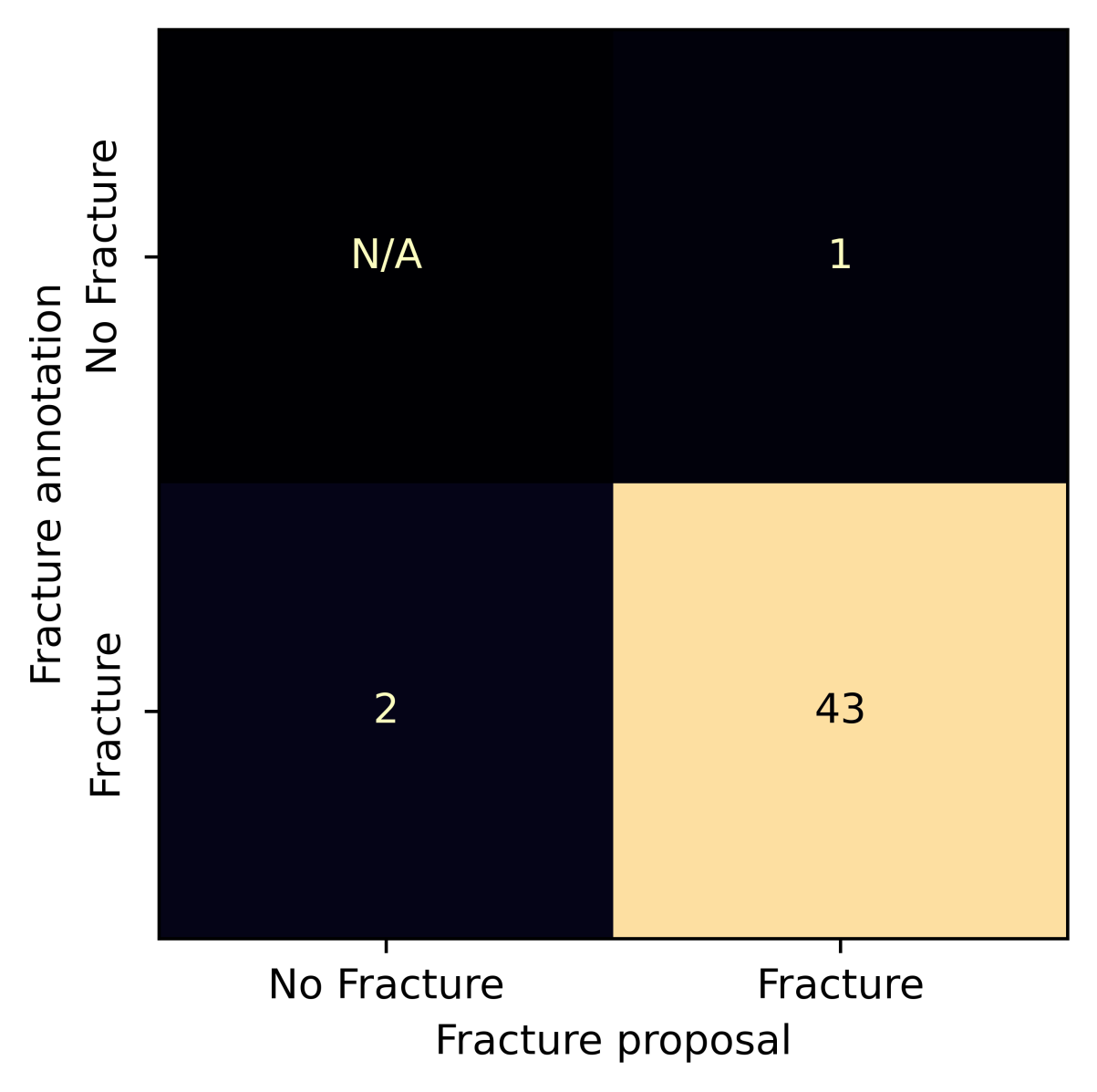


**Appendix Figure 3:** Results of mandibular fracture detection in a 2x2 table. A total of 45 fractures were annotated in 35 held-out test scans, of which two were not detected by JawFracNet (false-negative detection). Additionally, JawFracNet generated one erroneous fracture proposal (false-positive detection).

| **Scan** | **Fracture 1** | | **Fracture 2** | | **Fracture 3** | |
| --- | --- | --- | --- | --- | --- | --- |
|  | Confidence | Positive | Confidence | Positive | Confidence | Positive |
| 1 | 0.8104 | 1 | 0.8121 | 1 |  |  |
| 2 | 0.7798 | 1 | 0.7747 | 1 |  |  |
| 3 | 0.8223 | 1 | 0.7838 | 1 |  |  |
| 4 | -1 | 1 |  |  |  |  |
| 5 | 0.8041 | 1 |  |  |  |  |
| 6 | 0.8146 | 1 |  |  |  |  |
| 7 | 0.8074 | 1 |  |  |  |  |
| 8 | 0.8214 | 1 | 0.6861 | -1 |  |  |
| 9 | 0.8344 | 1 |  |  |  |  |
| 10 | 0.8242 | 1 | 0.8263 | 1 |  |  |
| 11 | 0.7940 | 1 |  |  |  |  |
| 12 | 0.8389 | 1 |  |  |  |  |
| 13 | 0.8323 | 1 | 0.8519 | 1 |  |  |
| 14 | 0.7836 | 1 |  |  |  |  |
| 15 | 0.8117 | 1 |  |  |  |  |
| 16 | 0.8204 | 1 |  |  |  |  |
| 17 | 0.7509 | 1 | 0.8347 | 1 |  |  |
| 18 | 0.8148 | 1 | 0.5041 | 1 |  |  |
| 19 | 0.8427 | 1 |  |  |  |  |
| 20 | 0.8156 | 1 |  |  |  |  |
| 21 | 0.7804 | 1 |  |  |  |  |
| 22 | 0.7857 | 1 |  |  |  |  |
| 23 | 0.8396 | 1 |  |  |  |  |
| 24 | 0.7938 | 1 |  |  |  |  |
| 25 | 0.8133 | 1 |  |  |  |  |
| 26 | 0.8446 | 1 |  |  |  |  |
| 27 | 0.7862 | 1 |  |  |  |  |
| 28 | 0.8337 | 1 | 0.8450 | 1 | 0.8219 | 1 |
| 29 | 0.8264 | 1 |  |  |  |  |
| 30 | 0.8385 | 1 |  |  |  |  |
| 31 | 0.8457 | 1 |  |  |  |  |
| 32 | 0.6283 | 1 |  |  |  |  |
| 33 | 0.8057 | 1 |  |  |  |  |
| 34 | 0.5237 | 1 |  |  |  |  |
| 35 | 0.6957 | 1 | -1 | 1 |  |  |

**Appendix Table 1:** Detailed results of JawFracNet for 35 held-out test scans. One to three fractures were annotated per scan and were matched to the model’s fracture proposals. For each match, the model’s confidence in the fracture proposal was determined as the mean segmentation probability. A failure to match a fracture annotation to any fracture proposal was denoted as “-1” in the Confidence column. Likewise, a failure to match a fracture proposal to any fracture annotation was denoted as “-1” in the Positive column.

## Model Facts label

**Description:** This supplementary file contains the Model Facts label (MFL) of JawFracNet, the proposed method in the article related to this file (Sendak et al. 2020). The inclusion of an MFL in the publication of an automated system involved in clinical decision-making facilitates the adoption of that system in clinical practice. The structured and clear format of an MFL allows for clinicians to quickly and reliably learn about the use cases and risk of an automated system.

| **Model Facts** | **Name:** JawFracNet | **Locale:** University Medical Center Hamburg-Eppendorf |
| --- | --- | --- |
| **Date:** 20^th^ March 2024 | | **Version:** 0.1 |
| **Summary**  This model uses a cone-beam computed tomography (CBCT) scan of (a part of) the oral and maxillofacial region to predict the location of mandibular fractures. It was developed in 2022 on behalf of Charité – Universitätsmedizin Berlin and was published online in 2023. | | |
| **Mechanism**   - **Outcome:** Non-displaced and displaced mandibular fractures - **Output:** Binary segmentation of the CBCT scan of fracture lines - **Target population:** All adolescent to adult patients > 16 years old with a suspected mandibular fracture - **Time of prediction:** Immediately following the acquisition of the CBCT scan - **Input data source:** CBCT scan of (a part of) the oral and maxillofacial region - **Input data type:** DICOM, NIfTI, MHA - **Training data:** UMC Hamburg-Eppendorf, 07/2012 - 09/2022 - **Model type:** Convolutional Neural Network | | |
| **Validation and performance**   \|  \| **AUC** \| **Sensitivity** \| **Precision** \| **Cohort type** \| **Cohort URL/DOI** \| \| --- \| --- \| --- \| --- \| --- \| --- \| \| **Local Retrospective** \| 0.956 \| 0.956 \| 0.978 \| Diagnostic \| **TBD** \| \| **External** \| **TBD** \| **TBD** \| **TBD** \| **TBD** \| **TBD** \| \| **Target Population** \| 0.956 \| 0.956 \| 0.978 \| Diagnostic \| **TBD** \| | | |
| **Uses and directions**   - **Benefits:** Automated assessment of mandibular fractures can reduce analysis time and diagnostic errors. - **Target population and use case:** When a patient with head trauma is first admitted to the department of oral and maxillofacial surgery, a cone-beam computed tomography (CBCT) scan is acquired and analyzed by the model to detect mandibular fractures in support of an oral and maxillofacial radiologist. - **General use:** This model is intended to be used in collaboration with an oral and maxillofacial surgeon or radiologist to improve the procedure of detecting mandibular fractures following head trauma. The medical professional will integrate clinical information, the fracture proposals of the model, and their own analysis of the CBCT scan to form a conclusion about the anatomical situation of the mandible, e.g. whether it is fractured. - **Appropriate decision support:** Disagreements between the model and medical professional can be resolved by a second independent assessment and joint discussion to come to a consensus. - **Before using this model:** Test the model prospectively on a diagnostic cohort that represents the target population for which the model will be used to validate it within a local setting. - **Safety and efficacy evaluation:** An external validation of the model on a diagnostic cohort from a different medical center is planned. | | |
| **Warnings**   - **Risks:** Clinicians using this model can still make diagnostic errors including missed fractures and misdiagnosed fractures, resulting in inappropriate treatment decisions and patient morbidity. - **Inappropriate settings:** The model was trained on scans with fractures isolated to the mandible and has not been validated for comminuted fractures or scans with fractures of other bones in the facial skeleton. - **Clinical rationale:** The model has no knowledge of regularly co-occurring fracture locations on contralateral sides of the mandible, so clinical experience can benefit in identifying fractures missed by the model. - **Inappropriate decision support:** A segmentation of a detected fracture may not be indicative of the extent of the fracture line and analysis of the extent of the fracture line must be conducted prior to treatment decision. - **Generalizability:** This model was primarily evaluated within the local setting of UMC Hamburg-Eppendorf. Do not use this model in an external setting without further validation. - **Discontinue use if:** Clinical staff raise concerns about utility of the model for the indicated use case or large, systematic changes occur at the data level (e.g. different CBCT scanner) that necessitates re-training of the model. | | |
| **Other information:**   - **Online availability:** <https://grand-challenge.org/algorithms/jawfracnet> - **Open-source code:** <https://github.com/nnistelrooij/jawfrac> - **For inquiries and additional information:** please email [Niels.vanNistelrooij@radboudumc.nl](mailto:Niels.vanNistelrooij@radboudumc.nl) | | |

## References

Jin L, Yang J, Kuang K, Ni B, Gao Y, Sun Y, Gao P, Ma W, Tan M, Kang H et al. 2020. Deep-learning-assisted detection and segmentation of rib fractures from ct scans: Development and validation of fracnet. EBioMedicine. 62(103106).

Sendak MP, Gao M, Brajer N, Balu S. 2020. Presenting machine learning model information to clinical end users with

model facts labels. npj Digital Medicine. 3:41.
