# Supplementary figures and images for: Detecting Mandible Fractures in CBCT Scans Using a 3-Stage Neural Network
Source: J Dent Res. 2024 Jun 24;103(13):1384–91. doi: 10.1177/00220345241256618 (PMC11633064; doi:10.1177/00220345241256618)

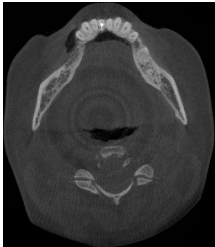

(a)

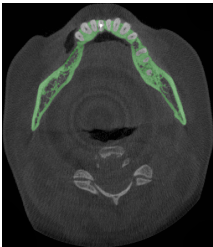

(b)

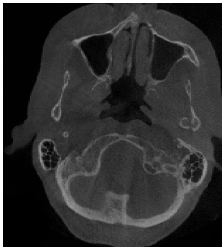

(c)

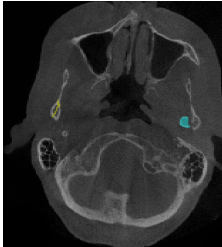

(d)

Supplement: sj-pdf-2-jdr-10.1177_00220345241256618 – Supplemental material for Detecting Mandible Fractures in CBCT Scans Using a 3-Stage Neural Network [file sj-pdf-2-jdr-10.1177_00220345241256618.pdf]

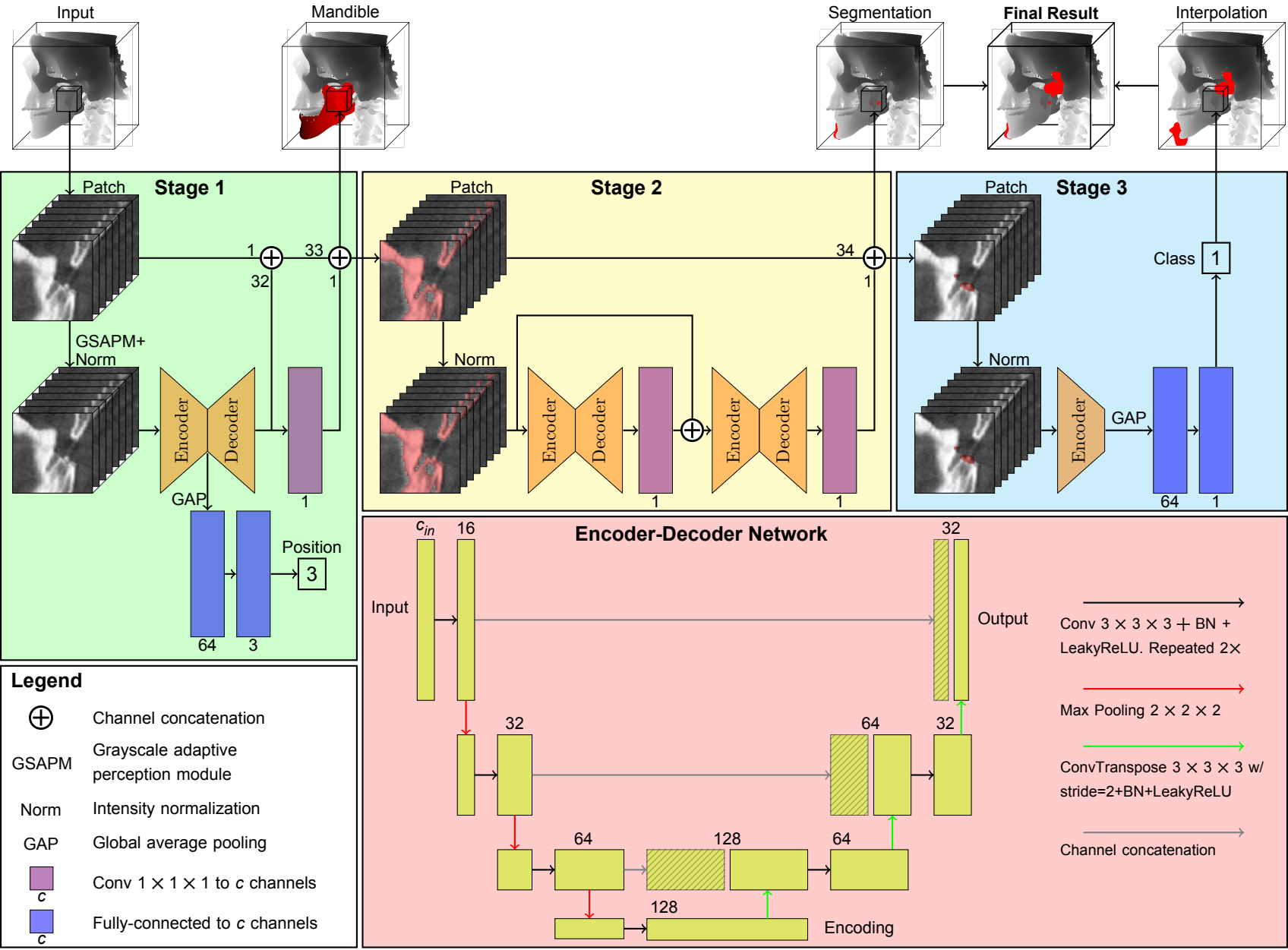

Supplement: sj-pdf-3-jdr-10.1177_00220345241256618 – Supplemental material for Detecting Mandible Fractures in CBCT Scans Using a 3-Stage Neural Network [file sj-pdf-3-jdr-10.1177_00220345241256618.pdf]

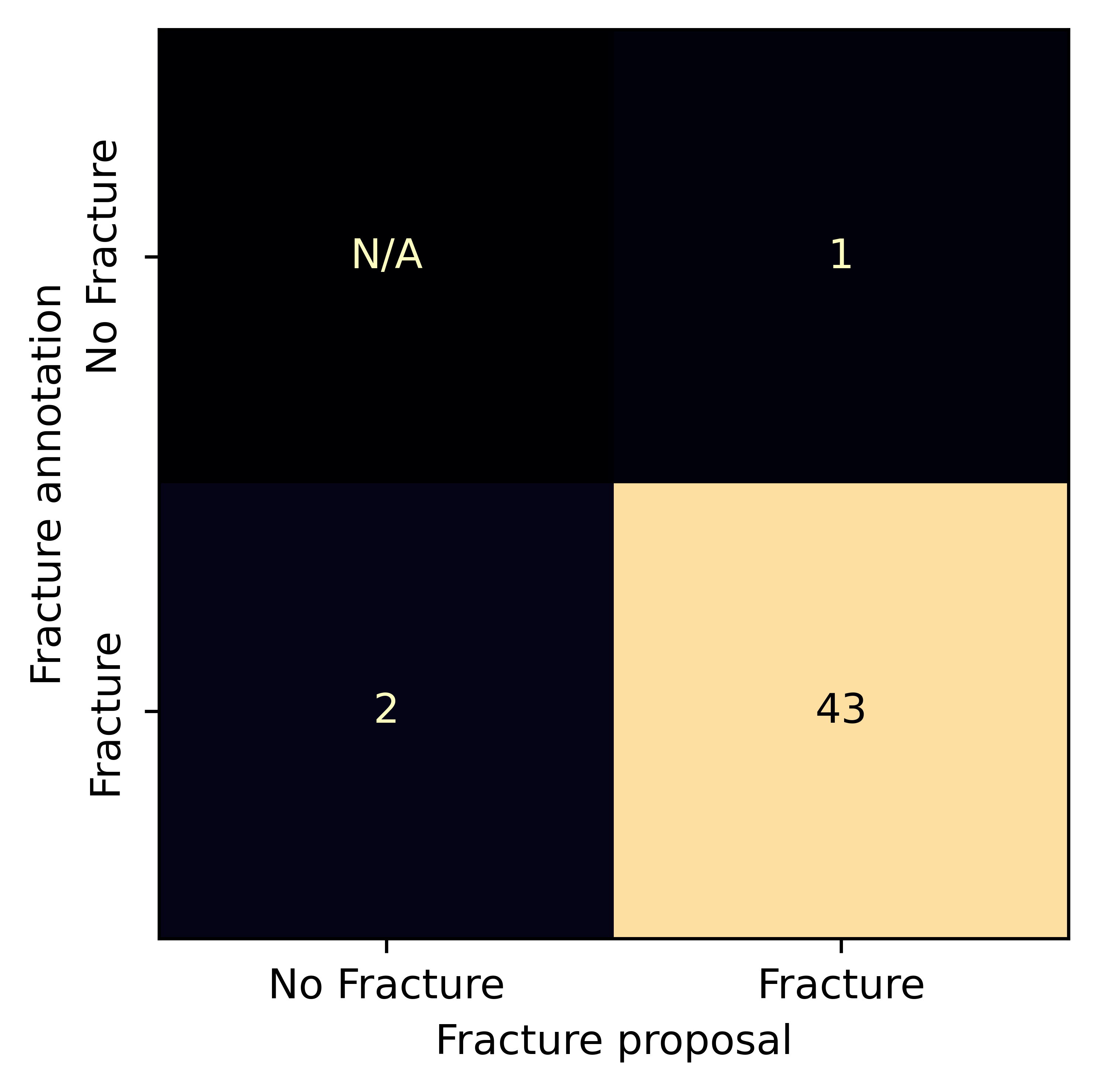

Supplement: sj-png-4-jdr-10.1177_00220345241256618 – Supplemental material for Detecting Mandible Fractures in CBCT Scans Using a 3-Stage Neural Network [file sj-png-4-jdr-10.1177_00220345241256618.png]
